# High CXCR3 on Leukemic Cells Distinguishes *IgHV*^mut^ from *IgHV*^unmut^ in Chronic Lymphocytic Leukemia: Evidence from CD5^high^ and CD5^low^ Clones

**DOI:** 10.1155/2020/7084268

**Published:** 2020-06-20

**Authors:** Gayane Manukyan, Tomas Papajik, Zuzana Mikulkova, Renata Urbanova, Veronika Smotkova Kraiczova, Jakub Savara, Milos Kudelka, Peter Turcsanyi, Eva Kriegova

**Affiliations:** ^1^Department of Immunology, Faculty of Medicine and Dentistry, Palacký University Olomouc and University Hospital, Olomouc, Czech Republic; ^2^Laboratory of Molecular and Cellular Immunology, Institute of Molecular Biology NAS RA, Yerevan, Armenia; ^3^Department of Hemato-Oncology, Faculty of Medicine and Dentistry, Palacký University and University Hospital, Olomouc, Czech Republic; ^4^Department of Computer Science, Faculty of Electrical Engineering and Computer Science, VSB-Technical University of Ostrava, Czech Republic

## Abstract

Despite the shared pattern of surface antigens, neoplastic cells in chronic lymphocytic leukemia (CLL) are highly heterogeneous in CD5 expression, a marker linked to a proliferative pool of neoplastic cells. To further characterize CD5^high^ and CD5^low^ neoplastic cells, we assessed the chemokine receptors (CCR5, CCR7, CCR10, CXCR3, CXCR4, CXCR5) and adhesion molecules (CD54, CD62L, CD49d) on the CD5^high^ and CD5^low^ subpopulations, defined by CD5/CD19 coexpression, in peripheral blood of CLL patients (*n* = 60) subgrouped according to the *IgHV* mutational status (*IgHV*^mut^, *n* = 24; *IgHV*^unmut^, *n* = 36). CD5^high^ subpopulation showed a high percentage of CXCR3 (*P* < 0.001), CCR10 (*P* = 0.001), and CD62L (*P* = 0.031) and high levels of CXCR5 (*P* = 0.005), CCR7 (*P* = 0.013) compared to CD5^low^ cells expressing high CXCR4 (*P* < 0.001). Comparing *IgHV*^mut^ and *IgHV*^unmut^ patients, high levels of CXCR3 on CD5^high^ and CD5^low^ subpopulations were detected in the *IgHV*^mut^ patients, with better discrimination in CD5^low^ subpopulation. Levels of CXCR3 on CD5^low^ subpopulation were associated with time to the next treatment, thus further confirming its prognostic value. Taken together, our analysis revealed higher CXCR3 expression on both CD5^high^ and CD5^low^ neoplastic cells in *IgHV*^mut^ with a better prognosis compared to *IgHV*^unmut^ patients. Contribution of CXCR3 to CLL pathophysiology and its suitability for prognostication and therapeutic exploitation deserves future investigations.

## 1. Introduction

Chronic lymphocytic leukemia (CLL) is a lymphoproliferative malignancy of clonally expanded heterogeneous pool of neoplastic B cells with aberrant expression of CD5 [1, 2], which are highly variably distributed between bone marrow, lymphoid organs, and peripheral blood. There is a growing body of evidence that proliferation of neoplastic cells plays a critical role in CLL pathogenesis [3–5], with the highest degree of proliferation being observed in the lymph nodes [6].

CD5, a marker normally present on T cells, acts as a repressor of B-cell receptor (BCR) signalling in CLL [7]. Proliferating, migrating CLL cells have been shown to preferentially express high levels of CD5, while the low levels of CD5 are associated with resting, circulating cells [8]. The overlapping BCR repertoires between CD5^high^ and CD5^low^ cells suggest a dynamic relationship of these two B-CLL cell subpopulations [2]. The inversed expression of CD5 and CXCR4 was used for the identification of fractions enriched in recently born/divided and older/quiescent CLL cells [8, 9]. It has been shown that CXCR4^dim^CD5^bright^ “proliferative” cells overexpress more “cell division” genes, while CXCR4^bright^CD5^dim^ “resting” cells express higher levels of “antiproliferative” genes, suggesting that the last subset could be a distinct self-renewing one from which all clonal members derive [8, 9].

Given the dynamic variability and heterogeneity of CD5 expression and its link to the proliferative pool of neoplastic cells, we aimed to further characterize the chemokine and adhesion molecule profile of CD5^high^ and CD5^low^ neoplastic clones using the novel CD5/CD19 model in peripheral blood of CLL patients. Being important for lymphocyte migration, we evaluated the expression of molecules linked to adhesion and extravasation (CD54, CD62L, CD49d), migration into lymph nodes (CCR7, CXCR3, CXCR5), homing lymphocytes to the bone marrow (CXCR4), and lymphocyte trafficking (CCR5, CCR10) in CD5^high^ and CD5^low^ neoplastic clones. Moreover, the differential expression pattern of chemokines and adhesion molecules on CD5^high^ and CD5^low^ clones was for the first time studied in two biologically and clinically distinct CLL subtypes defined by the abundance of somatic hypermutations affecting the Ig variable heavy-chain locus (*IgHV*), which markedly differ in their prognosis and response to the chemoimmunotherapy [10–12].

## 2. Materials and Methods

### 2.1. Patients

The patient cohort consisted of 60 patients with CLL, all diagnosed according to the IWCLL guidelines [13]. Patient subgroups were formed based on *IgHV* mutational status (*IgHV*^mut^, *n* = 24; *IgHV*^unmut^, *n* = 36). Clinical characteristics of CLL patients are shown in Table 1. All patients provided written informed consent for the use of peripheral blood for research purposes in accordance with the Declaration of Helsinki. The study was approved by the ethics committee of University Hospital and Palacký University Olomouc.

### 2.2. Flow Cytometry Analysis of Chemokine Receptors and Adhesion Molecules

Cells in whole blood were stained with optimal concentrations of monoclonal antibody combinations directed against the following surface antigens: CD45-PerCP/Cy5.5, CD5-PE, CD19-APC/Cy7, CD54-FITC, CD62L-APC, CD49d-PE/Cy7, CD183-FITC (CXCR3), CD184-APC (CXCR4), CD185-FITC (CXCR5), CD197-PE/Cy7 (CCR7), CD195-PE/Cy7 (CCR5), CCR10-APC (all BioLegend), as reported previously [14, 15]. Isotype-matched antibodies were used as negative controls. The analysis was performed on BD FACSCanto II (Becton Dickinson). Data acquisition was performed using BD FACSDiva software (Becton Dickinson). Flow cytometry data were analysed using the FlowJo vX0.7 software (Tree Star, Inc, San Carlos, CA). In all experiments, a minimum of 10,000 events was counted. Results are expressed as the percentage and median fluorescence intensity (MFI).

### 2.3. Identification of CD5^high^ and CD5^low^ Subpopulations

**Figure 1:**
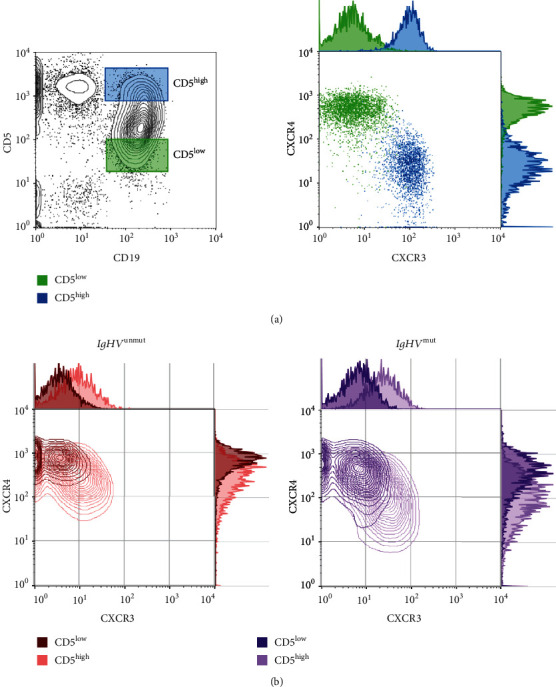
(a) Gating strategy for detection of CD5^high^ and CD5^low^ subpopulations within CD5+CD19+neoplastic cells and visualization of their CXCR3 and CXCR4 expression. (b) CXCR3 and CXCR4 expression on CD5^high^ and CD5^low^ subpopulations in *IgHV*^unmut^ and *IgHV*^mut^ patient—representative example.

Coexpression of CD5 and CD19, surface molecules essential for phenotypic characteristic of CLL cells, was used to discriminate between CD5^high^ and CD5^low^ subsets of CLL cells. Gating strategy for detection of CD5^high^ and CD5^low^ subpopulations and representative examples of CXCR3 and CXCR4 expression in *IgHV*^mut^ and *IgHV*^unmut^ patients are shown in Figure 1.

### 2.4. Chemotaxis Assay

Peripheral blood mononuclear cells (PBMC) were isolated using a Ficoll density gradient centrifugation; only samples containing more than 70% CLL cells in PBMC were chosen for the assay. Transmigration of CLL cells was assessed using polycarbonate Transwell inserts with 5-*μ*m pore size (Corning Costar). Briefly, the cells at 1 × 10exp 6/mL were applied to the upper chamber in RPMI-1640 containing 1% bovine serum albumin (BSA) in the presence or absence of CXCL11 (BioLegend). Filters were transferred into the lower wells containing RPMI-1640 with 1% BSA in the presence or absence of CXCL12 (BioLegend). After 3 hours at 37°C in 5% CO_2_, cells that migrated into the lower chambers were counted and analysed for CXCR3 and CXCR4 expression on BD FACSCanto II.

### 2.5. Statistical and Data Mining Analyses

Statistical analyses (Mann-Whitney **U**-test, paired Wilcoxon nonparametric test, Kruskal-Wallis test, 95% confidence intervals (CI), receiver operating characteristic (ROC) curves, the Kaplan-Meier curve, Spearman correlation analysis) were performed using the **R** statistical software package (http://www.r-project.org/). The multivariate patient similarity networks (PSNs) based on the nearest neighbour analysis [16, 17] were applied for the visualization of patient similarities of chemokine profiles. Correlation networks using LRNet algorithm [16] and Spearman's rank correlation coefficient were constructed and visualized to investigate the relationships between expression of individual molecules on CLL cells [18]. **P** values <0.05 were considered significant.

## 3. Results

### 3.1. CD5/CD19 Markers as Identifiers of CD5^high^ and CD5^low^ Cells

First, we identified CD5^high^ and CD5^low^ cells based on the CD5/CD19 model and compared it with the reported CD5^low^/CXCR4^high^ model [8, 9]. The CD5^high^ cells in the CD5/CD19 model corresponded to those in the CD5^high^/CXCR4^low^ model, and similarly CD5^low^ cells corresponded to those in the CD5^low^/CXCR4^high^ one (Figure S1A). High interindividual variability in the proportion of both closely related subpopulations of CD5^high^ and CD5^low^ cells was observed, irrespective of *IgHV* mutational status and other clinical characteristics (Figure S1B).

### 3.2. Differential Expression of Chemokine Receptors and Adhesion Molecules on CD5^high^ and CD5^low^ CLL Cells

The expression of CD54, CD62L, CD49d, CCR5, CCR7, CCR10, CXCR3, CXCR4, and CXCR5 was evaluated on CD5^high^ and CD5^low^ subpopulations (Table S1).

**Figure 2:**
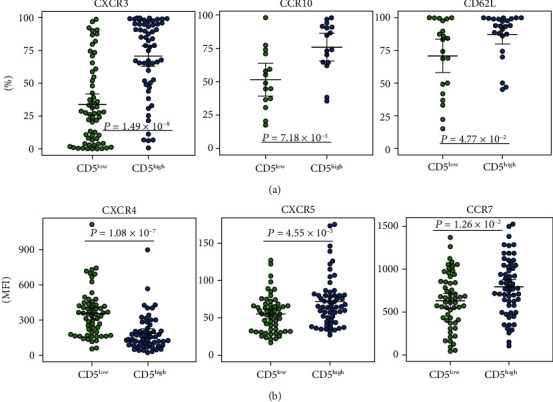
Distribution of (a) percentages of positive cells for CXCR3, CCR10, and CD62L and (b) expression (MFI) of CXCR4, CXCR5, and CCR7 on CD5^high^ and CD5^low^ subpopulations in patients with CLL. Group means are indicated by horizontal bars, error bars indicate 95% CI.

When analysing levels of studied markers (MFI) in CD5^high^ and CD5^low^ subpopulations, the CD5^high^ cells expressed higher levels of CXCR5 (*P* = 0.005) and CCR7 (*P* = 0.013) and lower levels of CXCR4 receptor (*P* < 0.001) than the population of CD5^low^ cells. Besides, more CD5^high^ cells were positive for CXCR3 (*P* < 0.001), CCR5 (*P* = 0.012), CCR10 (*P* = 0.007), and CD62L (*P* = 0.047) than CD5^low^ cells (Figure 2, Table S1).

### 3.3. Characterization of CD5^high^ and CD5^low^ CLL Cells in Patient Subgroups according to the *IgHV* Mutational Status

**Figure 3:**
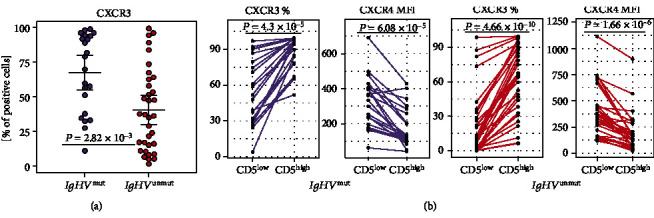
Distribution of CXCR3 and CXCR4 chemokines in *IgHV*^mut^ (violet) and *IgHV*^unmut^ (red) patients in (a) CLL cells as a whole, (b) CD5^high^, and CD5^low^ subpopulations in a paired analysis. The lines connect the values on CD5^high^ and CD5^low^ subpopulations in the same patient.

To characterize the CLL cells and their subpopulations in patient subgroups according to the *IgHV* mutational status, we compared the expression of studied markers on CLL cells as a whole and separately on CD5^high^ and CD5^low^ cells in CLL patients with *IgHV*^mut^ and *IgHV*^unmut^ status (Figure 3, Table S2, Table S3).

Of the studied markers, patients with *IgHV*^mut^ had a higher percentage of cells expressing CXCR3 (*P* = 0.003, Figure 3(a)) and CD62L (*P* = 0.003) in the whole population of CLL cells compared to those with *IgHV*^unmut^ status. When *IgHV*^mut^ and *IgHV*^unmut^ patients were evaluated separately, in both groups, a higher proportion of CLL cells positive on CXCR3 (in both *P* < 0.001) and lower expression of CXCR4 (in both *P* < 0.001) was observed on CD5^high^ subpopulation in comparison with CD5^low^ cells (Figure 3(b)). Similarly, when CD5^high^ and CD5^low^ cell populations were evaluated separately, the *IgHV*^mut^ group exhibited higher percentages of CXCR3 (in both *P* < 0.001), CD62L (in both *P* = 0.003) positive cells, as well as higher expression of CXCR5 (*P* < 0.001 and *P* = 0.011, respectively) in comparison with *IgHV*^unmut^ (Table S2, Table S3).

To exclude possible influence of the treatment on the studied parameters, we performed subanalysis on a cohort of untreated patients subdivided according to the *IgHV* mutational status, and we confirmed the differences for studied markers observed in the whole patient cohort (Figure S2A). Moreover, we did not observe significant differences in studied markers between *IgHV*^unmut^ patients untreated and those with treatment history (Figure S2B).

### 3.4. Correlation of CXCR3, CXCR4, and CXCR5 with CD5 Expression on CLL Cells

**Figure 4:**
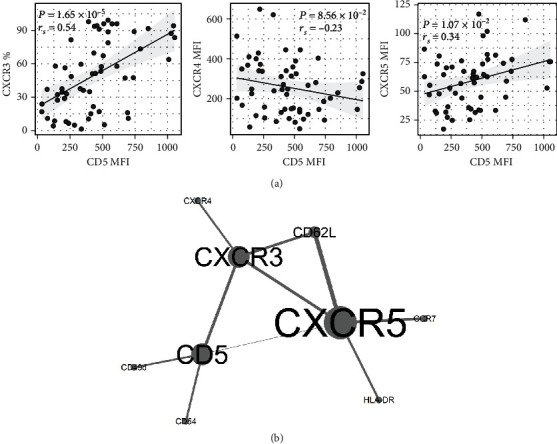
Correlation of CD5 expression with (a) percentages of CXCR3, levels (MFI) of CXCR4 and CXCR5 on CLL cells, and (b) studied chemokines on CLL cells assessed by the correlation network analysis.

On CLL cells, CD5 expression (MFI) positively correlated with the percentages and MFI of CXCR3 (*rs* = 0.54, *P* < 0.001 and *rs* = 0.54, *P* < 0.001) and MFI of CXCR5 (*rs* = 0.34, *P* = 0.010). There was a trend towards inverse correlation between CD5 and CXCR4 expression on the whole CLL subpopulation (*rs* = −0.23, *P*=0.086) (Figure 4(a)). Further information about correlations of CXCR3, CXCR4, and CXCR5 expression on CD5^high^ and CD5^low^ subpopulations is provided in the Supplementary file.

Regarding the relationship between CXCR3 and CXCR4, correlation analysis revealed a negative correlation between percentages as well as expression (MFI) of CXCR3 with CXCR4 on CLL cells (*rs* = −0.35, *P* = 0.009 and *rs* = −0.38, *P* = 0.006, respectively) (Figure S3). Network correlation analysis among studied chemokines and CD5 further confirmed a relationship and importance of CD5-CXCR3-CXCR5 axis on CLL cells (Figure 4(b)).

### 3.5. Migration Rate of CLL Cells in the Presence of CXCL11

**Figure 5:**
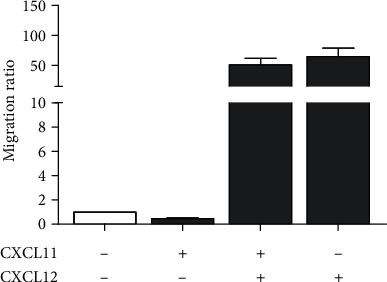
Cooperative effect of CXCL11 and CXCL12 on the migratory ability of CLL cells. CLL cells (*n* = 6), with/without pretreatment with 200 ng/mL CXCL11, were added to the upper chamber of Transwell inserts. 200 ng/mL CXCL12 was added to the medium in the bottom chamber, and migrated cells were counted after 3 hours by flow cytometry. Migration index was calculated as the ratio of cells that transmigrated through the insert in the chemokine-treated cells vs untreated ones (CXCR11-/CXCR12-).

To analyse the cooperative interplay between CXCR3 and CXCR4, we analysed the migratory ability of CXCL11-treated and untreated CLL cells towards chemokine CXCL12. The highest migration rates were observed for the CXCL11-untreated cells that migrated towards CXCL12 (*P* = 0.010) (Figure 5). When CLL cells were treated with CXCL11, their spontaneous migration as well as migration rate toward CXCL12 decreased (Figure 5).

### 3.6. CXCR3 On CLL Cells as a Prognostic Marker

**Figure 6:**
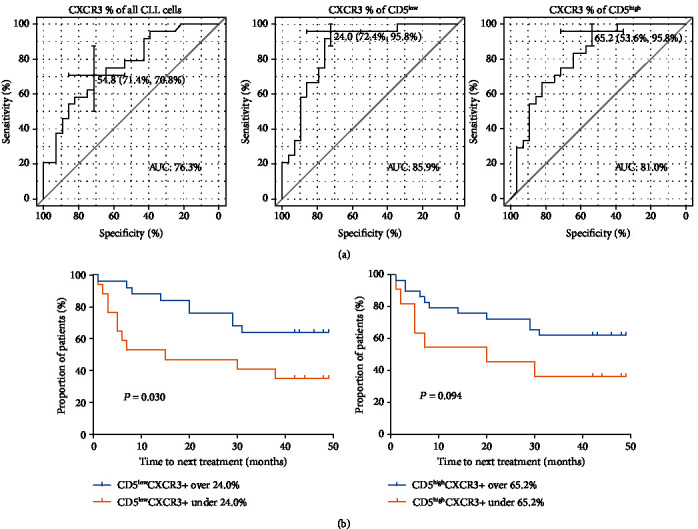
(a) Receiver operating characteristic (ROC) curves created for the percentage of CXCR3 positive all CLL cells, CD5^low^ and CD5^high^ cells, respectively, in patients with *IgHV*^mut^ and *IgHV*^unmut^ status. AUC: area under ROC curve. (b) Kaplan-Meier curves of time to next treatment in CLL patients according to the cut-off values of the percentage of CXCR3 on CD5^low^ and CD5^high^ cells.

To study the prognostic value of studied markers, we constructed ROC curves for CLL patients with favourable (*IgHV*^mut^) and unfavourable (*IgHV*^unmut^) prognostic groups. Among the studied markers, CXCR3 had the highest sensitivity and specificity on both CD5^high^ and CD5^low^ populations. Cut-off values for CXCR3 were 65.2%, 24.0%, and 54.8% for CD5^high^, CD5^low^, and CLL cells as a whole, respectively. Correspondingly, AUC reached values of 0.810, 0.859, and 0.763 on CD5^high^, CD5^low^, and CLL cells as a whole (Figure 6(a)). Kaplan-Meier curves using CXCR3 cut-off values showed the prognostic value of CXCR3 on CD5^low^ (*P* = 0.030) on time to the next treatment, calculated from the sampling time (Figure 6(b)). For the analysis, only patients with sufficient follow-up time were included.

### 3.7. Multivariate Patient Similarity Networks

**Figure 7:**
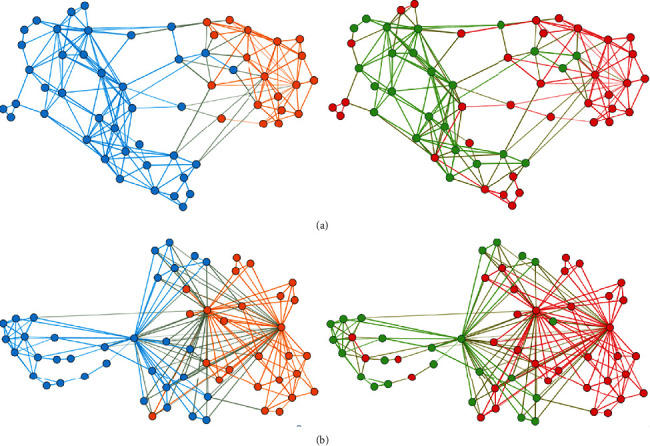
Patient similarity networks for the combination of chemokine expression (% of CXCR3, and MFI of CXCR4, CXCR5, CCR7) on (a) CD5^high^ and (b) CD5^low^ subpopulations in CLL patients. The nodes represent individual CLL patients, the lines connect the patients with high similarity in chemokine profiles. *IgHV*^mut^ patients are presented in green, *IgHV*^unmut^ patients in red. CXCR3 positive cells with cut-off <65% for CD5^high^ and <24% for CD5^low^, respectively, are orange, and CXCR3 positive cells with cut-off >65% for CD5^high^ and >24% for CD5^low^ are blue.

To gain more insights into CD5^high^ and CD5^low^ subpopulations in *IgHV*^mut^ and *IgHV*^unmut^ patients, we constructed the multivariate PSNs and performed their clustering based on the CXCR3, CXCR4, CXCR5, and CCR7 expression in enrolled CLL patients. Clusters with high CXCR3 include predominantly patients with *IgHV*^mut^ status, and vice versa clusters with low CXCR3 include predominantly patients with *IgHV*^unmut^ status. CXCR3 and other selected markers on CD5^low^ subpopulations better discriminate between patients with *IgHV*^mut^ and *IgHV*^unmut^ subgroups than markers on CD5^high^ (Figure 7). For clustering and distribution of chemokine expression in particular clusters and corresponding expression patterns, see the Supplementary file (Figure S4).

## 4. Discussion

There is a growing body of evidence that CLL neoplastic cells are composed of subpopulations of cells that differ in their biological function [1, 2, 8, 19, 20]. Our study revealed differences in the expression of molecules linked to adhesion, extravasation, migration, and homing, on CD5^high^ and CD5^low^ CLL populations, defined using the CD5/CD19 coexpression model, in the patient subgroups according to the *IgHV* mutational status.

In our CLL cohort, we observed high expression levels of CXCR3, CXCR5, CCR10, and CD62L on CD5^high^ cells and high CXCR4 on CD5^low^ cells, which is in line with a previous study [8]. Interestingly, our data showed that CXCR3 better discriminates both CD5^high^ and CD5^low^ cell populations than CXCR4, a key chemokine receptor involved in migration of CLL cells to the supportive lymphoid tissues [1, 21, 22]. Moreover, our study revealed differences in expression pattern between patient subgroups according to the *IgHV* mutational status, a key prognostic predictor of overall survival and treatment-response duration [10–12]. The most prominent were the differences between the percentages of CXCR3 on CD5^low^ cell subpopulation between *IgHV*^mut^ and *IgHV*^unmut^ statuses. Moreover, our results revealed that CXCR3 on CD5^low^ cells has the best prognostic utility in discriminating patients with *IgHV*^mut^ and *IgHV*^unmut^ status, and correspondingly the patients with favourable or poor prognosis. This observation contributes to further characterization of *IgHV*^mut^ and *IgHV*^unmut^ mutation statuses, known to differ in gene expression [23], methylation status [24], and the mutational landscape [25].

Recently, CXCR3 has been found as a marker of independent prognostic significance in CLL [26]. High CXCR3 expression and CXCR3/CXCR4 ratio delineated patients with a significantly better clinical course, as opposed to patients with low CXCR3 and high CXCR4 expression [26, 27]. To date, there is no clear understanding of how CXCR3 influences the pathogenesis of CLL. A formation of CXCR3-CXCR4 heteromers and a negative binding cooperativity between CXCR3 and CXCR4 at the cell surface was reported on CLL [26] and HEK293T [28] cells. Importantly, the CXCR3-CXCR4 heteromerization has been shown to alter the ligand-binding kinetics: CXCR3 and CXCR4 agonists have been proved to inhibit each other's equilibrium binding on membranes and specifically accelerate dissociation of CXCL12 from CXCR4 [28]. The negative impact of CXCR3 stimulation by its ligands CXCL9, CXCL10, and CXCL11 was shown to be highly specific for CXCR4-induced migration and resulted in reduced CXCR4-CXCL12 chemotaxis [26], as we also confirmed by the migration experiment in our patients. Given the critical role of CXCR4-CXCL12 axis in migration of CLL cells between blood and supportive lymphoid tissues in CLL, the formation of CXCR3-CXCR4 heterodimers on CLL cells and its consequences may significantly reduce the migration of CLL cells [1, 21, 22]. Since the proportion of CXCR3-CXCR4 heteromers is relative to homomers of both receptors [28], low levels of CXCR3 on CLL cells, observed in our patients with mutated *IgHV* status, might not be sufficient to abrogate migration of CLL cells driven by CXCR4. Contribution of CXCR3 to better prognosis and attenuation of CLL cell migration deserves future investigations.

Our study introduced a simple model based on CD5/CD19 coexpression for studying the CLL subpopulations. Given comparable data to a study using markers CD5/CXCR4 [8], CD5/CD19 coexpression might represent a combination of markers capable of reflecting biological differences between CLL clones, an assumption that needs to be verified in future studies. Moreover, the fact that the subpopulation of CLL cells in the CD5/CD19 model correspond to those in the CD5/CXCR4 model suggests that CD5 may play a more important pathogenic role in CLL than previously recognized. The precise function of CD5 in the interactions of immune cells remains unclear, especially on CLL cells. It was shown that this molecule negatively regulates B1 cell activation and activation-induced cell death [29, 30]. There is growing evidence of several pools of leukemia cells present in CLL, including circulating cell cycle arrested CLL cells expressing preferably low levels of CD5, and migrated activated cells that express high levels of CD5 and that are driven to proliferate via signals from the lymphoid tissue microenvironment [2, 8, 31, 32]. Despite this, CD5 positive cells were shown to have a longer lifespan than CD5 negative cells [29]. Whether CD5 contributes also to the recirculating capacity of CLL cells and their differential proliferative potential deserves further investigations. A deeper understanding of the CD5 role in CLL clones' biology may permit potentiation of current immunotherapeutic strategies.

The study has several limitations. First, we analysed a diagnostic real-world CLL cohort of patients sampled at different time points and treatment regimes. Second, our exploratory study should be followed by functional investigations on the role of CXCR3 on CD5^high^ and CD5^low^ cell populations in future studies. Despite these limitations, we believe that our novel model for distinguishing between proliferative and resting fractions of neoplastic cells and first study on characteristics of CLL subpopulations in CLL patients with different *IgHV* mutational statuses highlights the critical contribution of chemokine receptors to the disease outcome in CLL.

In summary, we present for the first time the marked differences in expression of chemokine receptor CXCR3 on CD5^high^ and CD5^low^ cell populations in patients with different *IgHV* mutational statuses. The wide presence of CXCR3 marker on CLL cells appears to portend a favourable prognosis, thus further supporting its potential as a prognostic marker. Understanding the pathological relevance of CD5^high^ and CD5^low^ cell subsets, their characteristics and phenotypes may likely broaden our understanding of CLL pathology as well as reveal novel therapeutic avenues.

## Figures and Tables

**Table 1:** Patient demographic and clinical characteristics.

Parameter	CLL (*n* = 60)
Age, years, median (min-max)	67 (50-86)
Gender (male/female)	35/25
Binet stage (A/B/C)	26/23/11
*IgHV* gene mutational status∗ (mutated/unmutated)	24/36
Genetics	
11q-/17p-	13/6
N/O	6/46
Not determined	2
Follow-up time months (mean, min–max)	53 (0-160)
Treatment history (yes/no)	29/31
Time of last treatment in treated patients (in respect to the sampling time) months (mean, min–max)	23 (1-61)
Time to the next treatment (in respect to the sampling time) months (mean, min–max)	29 (0-49)
CLL cells in peripheral blood	
Percentage, mean (95% CI)	69.3 (62.3-76.3)
Absolute number (×10^9^/L), mean (95% CI)	49.1 (32.3-65.9)

∗*IgHV* mutational status was defined as follows: *IgHV*^unmut^ with a cut-off of 2% deviation or >98% sequence identity to germline in the *IgHV* sequence (13). 11q- and 17p-: any FISH or karyotypic abnormality involving 11q or 17p; N: no detectable cytogenetic aberration by FISH; O: other cytogenetic abnormality (excluding 11q- or 17p-).

## Data Availability

The data used to support the findings of this study are available from the corresponding author upon request.

## Supplementary Materials

Supplementary materials Table S1: percentage of cells positive for studied chemokine receptors and adhesion molecules and their surface expression (MFI) on CD5^low^ and CD5^high^ subpopulations in enrolled patients. Table S2: percentage of cells positive for studied chemokine receptors and adhesion molecules on CD5^low^ and CD5^high^ subpopulations in CLL patients subdivided according to the *IgHV*^unmut^ and *IgHV*^mut^ status. Table S3: expression (MFI) of studied chemokine receptors and adhesion molecules on CD5^low^ and CD5^high^ subpopulations in *IgHV*^unmut^ and *IgHV*^mut^ patients. Figure S1: analysis of CLL cell subpopulations. Figure S2: distribution of cells positive for CXCR3, CXCR4, and CXCR5 on CD5^high^ and CD5^low^ subpopulations between untreated *IgHV*^mut^ and *IgHV*^unmut^ CLL patients and CLL patients with *IgHV*^unmut^ with/without treatment history. Figure S3: correlation analysis between percentages and MFI of CXCR3 and CXCR4 on CLL cells. Figure S4: patient similarity network based on levels of CXCR3, CXCR4, CRCR5, and CCR7, and distribution of chemokine expression in individual clusters.
