# Supplementary material for: High CXCR3 on Leukemic Cells Distinguishes IgHVmut from IgHVunmut in Chronic Lymphocytic Leukemia: Evidence from CD5high and CD5low Clones
Source: J Immunol Res. 2020 Jun 20;2020:7084268. doi: 10.1155/2020/7084268 (PMC7322588; doi:10.1155/2020/7084268)
Supplement: Supplementary materials — Table S1: percentage of cells positive for studied chemokine receptors and adhesion molecules and their surface expression (MFI) on CD5low and CD5high subpopulations in enrolled patients. Table S2: percentage of cells positive for studied chemokine receptors and adhesion molecules on CD5low and CD5high subpopulations in CLL patients subdivided according to the IgHVunmut and IgHVmut status. Table S3: expression (MFI) of studied chemokine receptors and adhesion molecules on CD5low and CD5high subpopulations in IgHVunmut and IgHVmut patients. Figure S1: analysis of CLL cell subpopulations. Figure S2: distribution of cells positive for CXCR3, CXCR4, and CXCR5 on CD5high and CD5low subpopulations between untreated IgHVmut and IgHVunmut CLL patients and CLL patients with IgHVunmut with/without treatment history. Figure S3: correlation analysis between percentages and MFI of CXCR3 and CXCR4 on CLL cells. Figure S4: patient similarity network based on levels of CXCR3, CXCR4, CRCR5, and CCR7, and distribution of chemokine expression in individual clusters. [file 7084268.f1.docx]

**Supplementary File**

**High CXCR3 on leukemic cells distinguishes *IgHV*^mut^ from *IgHV*^unmut^ in chronic lymphocytic leukemia: evidence from CD5^high^ and CD5^low^ clones**

Gayane Manukyan^1,4^, Tomas Papajik^2^, Zuzana Mikulkova^1^, Renata Urbanova^2^, Veronika Smotkova Kraiczova^1^, Jakub Savara^3^, Milos Kudelka^3^, Peter Turcsanyi^2^, Eva Kriegova^1#^

**SUPPLEMENTARY FILE CONTENT**

**Tables**

**Table S1.** Percentage of cells positive for studied chemokine receptors and adhesion molecules and their surface expression (MFI) on CD5^low^ and CD5^high^ subpopulations in enrolled patients.

**Table S2.** Percentage of cells positive for studied chemokine receptors and adhesion molecules on CD5^low^ and CD5^high^ subpopulations in CLL patients subdivided according to the *IgHV*^unmut^ and *IgHV*^mut^ status.

**Table S3.** Expression (MFI) of studied chemokine receptors and adhesion molecules on CD5^low^ and CD5^high^ subpopulations in *IgHV*^unmut^ and *IgHV*^mut^ patients.

**Figures**

**Figure S1.** Analysis of CLL cell subpopulations.

**Figure S2.** Distribution of cells positive for CXCR3, CXCR4 and CXCR5 on CD5^high^ and CD5^low^ subpopulations between untreated *IgHV*^mut^ and *IgHV*^unmut^ CLL patients and CLL patients with IgHV^unmut^ with/without treatment history.

**Figure S3.** Correlation analysis between percentages and MFI of CXCR3 and CXCR4 on CLL cells.

**Figure S4.** Patient similarity network based on levels of CXCR3, CXCR4, CRCR5, and CCR7, and distribution of chemokine expression in individual clusters.

**Table S1. (A)** Percentage of cells positive for studied chemokine receptors and adhesion molecules and **(B)** their surface expression (MFI) on CD5^low^ and CD5^high^ subpopulations in enrolled patients.

| *Marker* | *Mean (95% CI)* | | *FC^#^* | *P-value* |
| --- | --- | --- | --- | --- |
|  | **CD5^low^** | **CD5^high^** |  |  |
| CD5 (MFI) | 273 (248-298) | 754 (702-806) | 2.38 | 5.17 × 10^-21^ |
| **(A) Percentage of positive cells** |  |  |  |  |
| CXCR3 | 33.8 (25.8-41.9) | 70.6 (62.9-78.3) | 2.96 | 1.49 × 10^-8^ |
| CXCR4 | 96.8 (95.8-97.8) | 91.8 (88.8-94.8) | 0.98 | 2.81 × 10^-3^ |
| CXCR5 | 91.7 (88.3-95.0) | 97.2 (96.0-98.4) | 1.02 | 3.18 × 10^-3^ |
| CCR5 | 0.83 (0.01-1.65) | 2.95 (0.51-5.39) | 3.74 | 1.20 × 10^-2^ |
| CCR7 | 98.2 (97.0-99.4) | 99.3 (98.6-100) | 1.00 | 2.71 × 10^-2^ |
| CCR10 | 51.5 (39.2-63.8) | 75.9 (65.5-86.2) | 1.62 | 7.18 × 10^-3^ |
| CD54 | 35.3 (22.1-48.5) | 40.0 (26.8-53.1) | 1.19 | 0.654 |
| CD62L | 70.8 (57.9-83.6) | 87.2 (79.9-94.5) | 1.13 | 0.047 |
| CD49d | 29.1 (10.9-47.4) | 33.3 (15.8-50.7) | 0.96 | 0.768 |
| **B) Expression (MFI)** |  |  |  |  |
| CXCR3 | 25.5 (22.8-28.1) | 48.1 (40.0-56.2) | 1.73 | 6.02 × 10^-8^ |
| CXCR4 | 356 (304-409) | 188 (149-228) | 0.39 | 1.08 × 10^-7^ |
| CXCR5 | 55.4 (48.7-62.1) | 71.6 (62.9-80.4) | 1.18 | 4.55 × 10^-3^ |
| CCR5 | 30.2 (26.3-34.1) | 26.3 (25.6-26.9) | 0.97 | 0.202 |
| CCR7 | 633 (548-718) | 795 (706-883) | 1.21 | 1.26 × 10^-2^ |
| CCR10 | 30.9 (28.5-33.3) | 44.7 (37.9-51.5) | 1.39 | 7.53 × 10^-4^ |
| CD54 | 17.3 (16.0-18.5) | 19.1 (17.3-20.9) | 1.06 | 0.163 |
| CD62L | 98.2 (65.6-131) | 196 (127-265) | 1.48 | 3.10 × 10^-2^ |
| CD49d | 91.7 (56.0-127) | 66.1 (43.1-89.1) | 0.78 | 0.304 |

*^#^*FC (Fold Change) between group medians

CI (confidence interval)

**Table S2.** Percentage of cells positive for studied chemokine receptors and adhesion molecules on **(A)** CD5^low^ and **(B)** CD5^high^ subpopulations in CLL patients subdivided according to the *IgHV*^unmut^ and *IgHV*^mut^ status.

| *Marker* | *Mean (95% CI)* | | *FC#* | *P-value* |
| --- | --- | --- | --- | --- |
| **(A) CD5^low^ population** | ***IgHV^unmut^*** | ***IgHV^mut^*** |  |  |
| CD5 (MFI) | 274 (238-309) | 276 (234-318) | 0.93 | 0.993 |
| CXCR3 | 21.3 (12.0-30.7) | 53.6 (41.3-65.9) | 4.71 | 3.29 × 10^-5^ |
| CXCR4 | 96.9 (95.6-98.2) | 96.6 (94.8-98.3) | 0.99 | 0.931 |
| CXCR5 | 89.2 (84.4-94.0) | 98.2 (97.0-99.4) | 1.06 | 5.82 × 10^-5^ |
| CCR5 | 0.93 (-0.12-1.97) | 0.43 (-0.79-1.64) | 0.31 | 1.000 |
| CCR7 | 98.4 (96.9-99.9) | 99.7 (99.3-100) | 1.01 | 0.093 |
| CCR10 | 47.1 (35.3-58.8) | 69.4 (-7.43-146) | 1.58 | 0.295 |
| CD54 | 42.9 (18.6-67.2) | 27.8 (9.45-46.2) | 0.49 | 0.400 |
| CD62L | 56.5 (37.3-75.8) | 88.8 (76.5-101) | 1.66 | 2.88 × 10^-3^ |
| CD49d | 46.1 (11.6-80.6) | 14.4 (-6.83-35.7) | 0.15 | 0.315 |
| **(B) CD5^high^ population** | ***IgHV^unmut^*** | ***IgHV^mut^*** |  |  |
| CD5 (MFI) | 779 (709-848) | 719 (631-806) | 0.92 | 0.119 |
| CXCR3 | 60.4 (49.4-71.5) | 87.7 (81.5-94.0) | 1.45 | 5.97 × 10^-4^ |
| CXCR4 | 89.8 (84.8-94.8 | 93.8 (90.6-97.1) | 0.99 | 0.487 |
| CXCR5 | 96.6 (95.2-97.9) | 99.4 (99.1-99.7) | 1.02 | 2.42 × 10^-4^ |
| CCR5 | 3.36 (0.33-6.39) | 1.17 (-0.85-3.20) | 0.45 | 0.611 |
| CCR7 | 99.7 (99.5-99.9 | 99.9 (99.7-100) | 1.01 | 0.036 |
| CCR10 | 74.5 (62.2-86.8) | 81.9 (41.2-123) | 1.15 | 0.800 |
| CD54 | 45.6 (24.2-66.9) | 33.6 (13.5-53.8) | 0.73 | 0.295 |
| CD62L | 81.7 (69.9-93.5) | 95.7 (90.4-101) | 1.12 | 2.81 × 10^-3^ |
| CD49d | 50.9 (20.8-80.9) | 18.8 (-2.90-40.5) | 0.06 | 0.230 |

*^#^*FC (Fold Change) between group medians

CI (confidence interval)

**Table S3.** Expression (MFI) of studied chemokine receptors and adhesion molecules on **(A)** CD5^low^ and **(B)** CD5^high^ subpopulations in *IgHV*^unmut^ and *IgHV*^mut^ patients.

| *Marker* | *Mean (95% CI)* | | *FC#* | *P-value* |
| --- | --- | --- | --- | --- |
| **(A) CD5^low^ population** | ***IgHV^unmut^*** | ***IgHV^mut^*** |  |  |
| CD5 | 274 (238-309) | 276 (234-318) | 0.93 | 0.993 |
| CXCR3 | 22.2 (19.3-25.1) | 29.9 (24.8-35.0) | 1.19 | 8.11 × 10^-4^ |
| CXCR4 | 383 (301-464) | 313 (248-378) | 0.90 | 0.503 |
| CXCR5 | 47.4 (38.8-56.0) | 68.1 (57.7-78.5) | 1.57 | 8.14 × 10^-4^ |
| CCR5 | 31.3 (26.3-36.3) | 26.4 (22.8-30.1) | 0.89 | 0.351 |
| CCR7 | 593 (488-699) | 755 (608-903) | 1.12 | 0.093 |
| CCR10 | 30.8 (28.1-33.4) | 31.7 (-16.6-80.0) | 1.05 | 0.791 |
| CD54 | 17.4 (15.0-19.8) | 17.2 (15.4-19.0) | 1.05 | 0.789 |
| CD62L | 69.1 (32.3-106) | 137 (81.0-193) | 2.56 | 0.034 |
| CD49d | 72.6 (27.2-118) | 115 (52.1-178) | 1.10 | 0.315 |
| **(B) CD5^high^ population** | ***IgHV^unmut^*** | ***IgHV^mut^*** |  |  |
| CD5 | 779 (709-848) | 719 (631-806) | 0.92 | 0.119 |
| CXCR3 | 41.4 (30.3-52.6) | 59.7 (46.5-72.9) | 1.98 | 2.61 × 10^-3^ |
| CXCR4 | 192 (129-255) | 176 (126-225) | 0.80 | 0.874 |
| CXCR5 | 66.2 (52.4-80.0) | 81.2 (69.9-92.5) | 1.30 | 0.011 |
| CCR5 | 26.4 (25.7-27.1) | 25.6 (24.9-26.4) | 0.97 | 0.177 |
| CCR7 | 750 (631-871) | 911 (777-1045) | 1.15 | 0.089 |
| CCR10 | 44.8 (37.0-52.7) | 44.2 (8.15-80.2) | 0.90 | 1.000 |
| CD54 | 19.8 (16.7-22.9) | 18.6 (16.0-21.3) | 0.95 | 0.441 |
| CD62L | 118 (58.0-179) | 279 (163-395) | 2.61 | 0.018 |
| CD49d | 70.0 (44.6-95.4) | 65.5 (22.7-108) | 0.40 | 0.497 |

FC# (Fold Change) between group medians

95% CI (confidence interval)

**Figure S1.** Analysis of CLL cell subpopulations. **(A)** Comparative analysis of CD5^high^ and CD5^low^ cells defined by CD5/CD19 and CD5/CXCR4 markers – representative example. **(B)** Representative density plots showing heterogeneity of CLL cells defined by CD5 and CD19 markers CLL in three patients (P1-P3): P1-equal proportions of CD5^high^ and CD5^low^ populations; P2-predominance of CD5^low^ cells; and P3- the predominance of CD5^high^ cells.

**
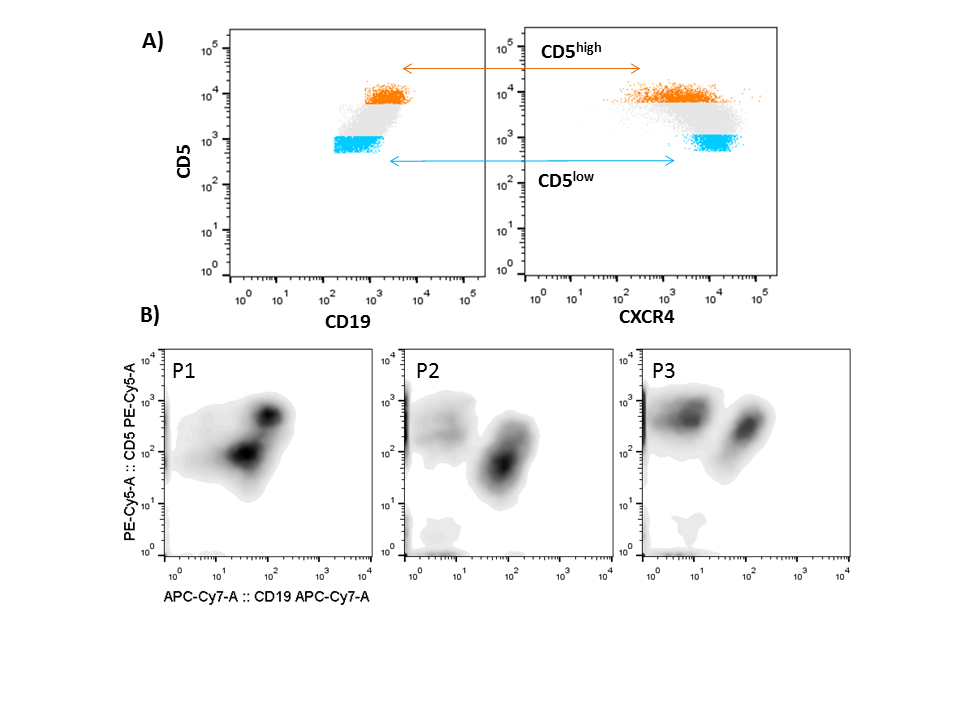
**

**Figure S2.** Distribution of cells positive for CXCR3, CXCR4 and CXCR5 on CD5^high^ and CD5^low^ subpopulations between **(A)** only untreated *IgHV^mut^* and *IgHV^unmut^* CLL patients and **(B)** CLL patients with *IgHV^unmut^* with/without treatment history. Group means are indicated by horizontal bars, error bars indicate 95% CI; *P* values for differences between two groups are stated.

**
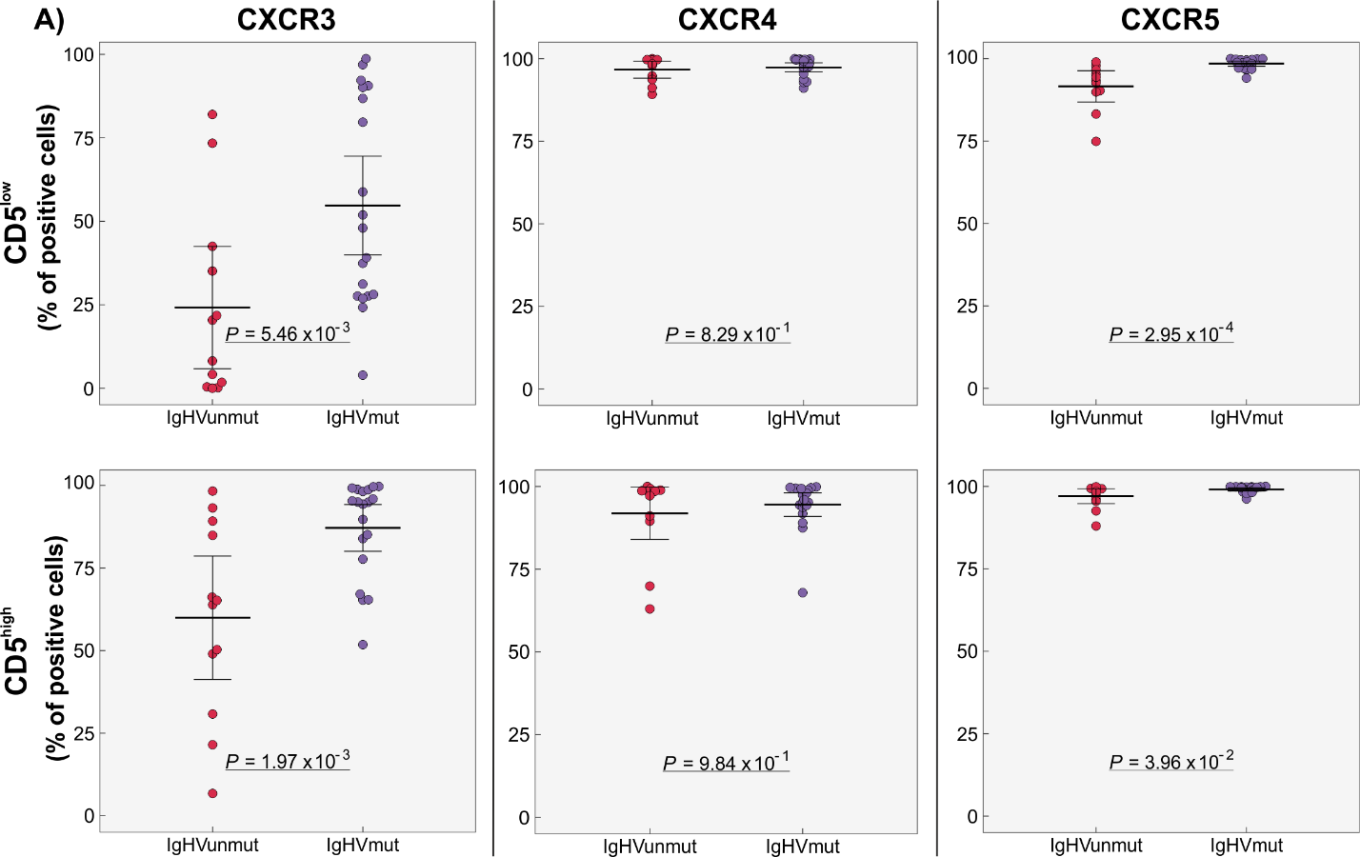
**

**B)**

**
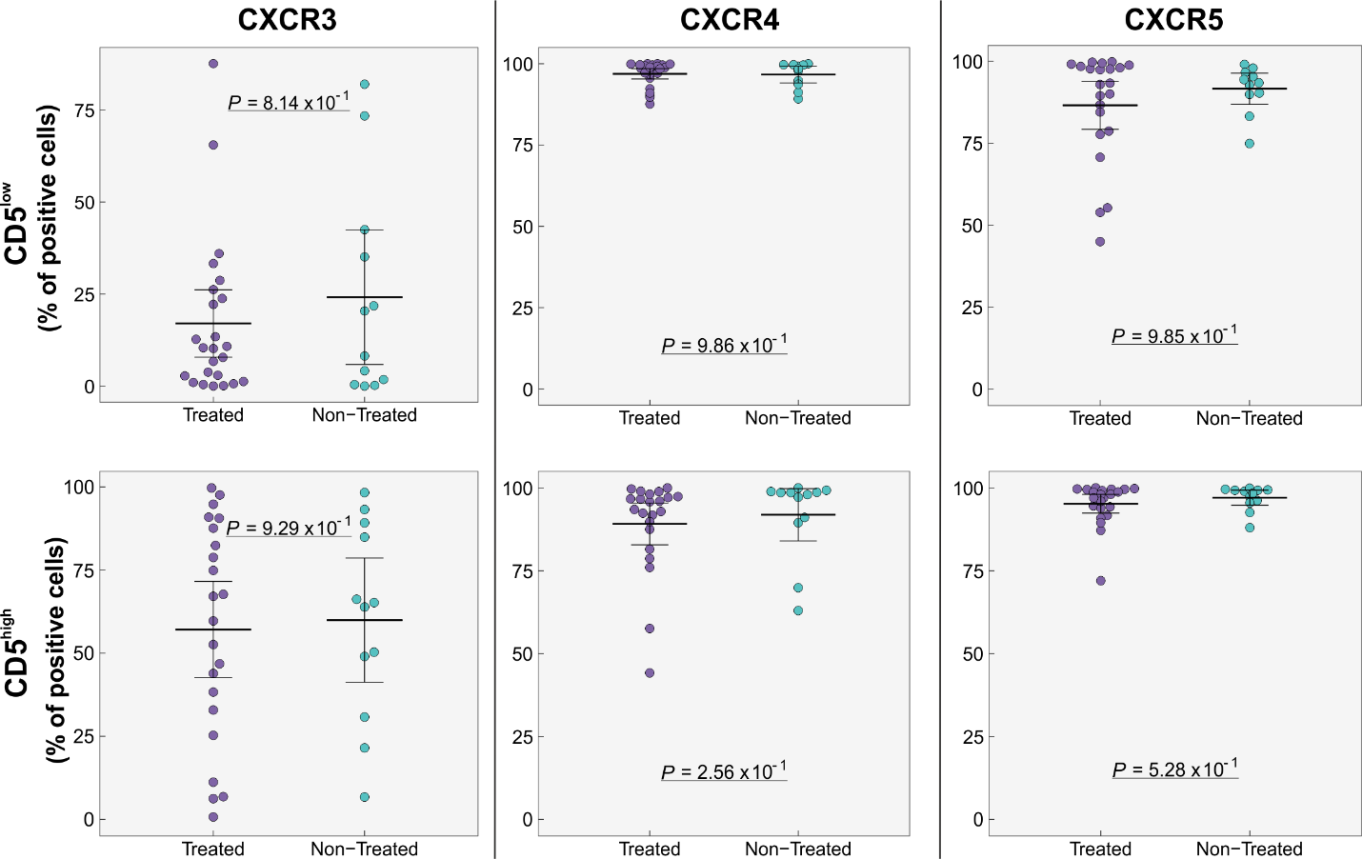
**

### *Correlation of* *CXCR3, CXCR4 and CXCR5 expression on CD5^high^ and CD5^low^ CLL cells*

Regarding the CD5 subpopulations, the percentage and expression (MFI) of CXCR3 positively correlated with the MFI of CD5^high^ cells (r_s_=0.47, *P*<0.001 and r_s_=0.54, *P*<0.001, respectively) and with the MFI of CD5^low^ cells (r_s_=0.43, *P*<0.001 and r_s_=0.46, *P*<0.001, respectively). Similar results were observed for correlation of CXCR5 expression with MFI of CD5^high^ cells (r_s_=0.32, *P=*0.014) and MFI of CD5^low^ cells (r_s_=0.34, *P*=0.007. In contrast to CXCR3 and CXCR5, a significant correlation of CD5 expression with MFI of CXCR4 on both CD5^high^ and CD5^low^ subpopulations was not observed ( r_s_=-0.07, *P*=0.601 and r_s_=0.10, *P=*0.449, respectively). Of studied adhesive molecules, the percentage of CLL cells negatively correlated with the percentage and expression (MFI) of CD62L (rs=-0.44, P=0.031 and rs=-0.47, P=0.019, respectively) and expression of CD54 (rs=-0.51, P=0.010).

**Figure S3.** Correlation analysis between **(A)** percentages and **(B)** MFI of CXCR3 and CXCR4 on CLL cells.

**
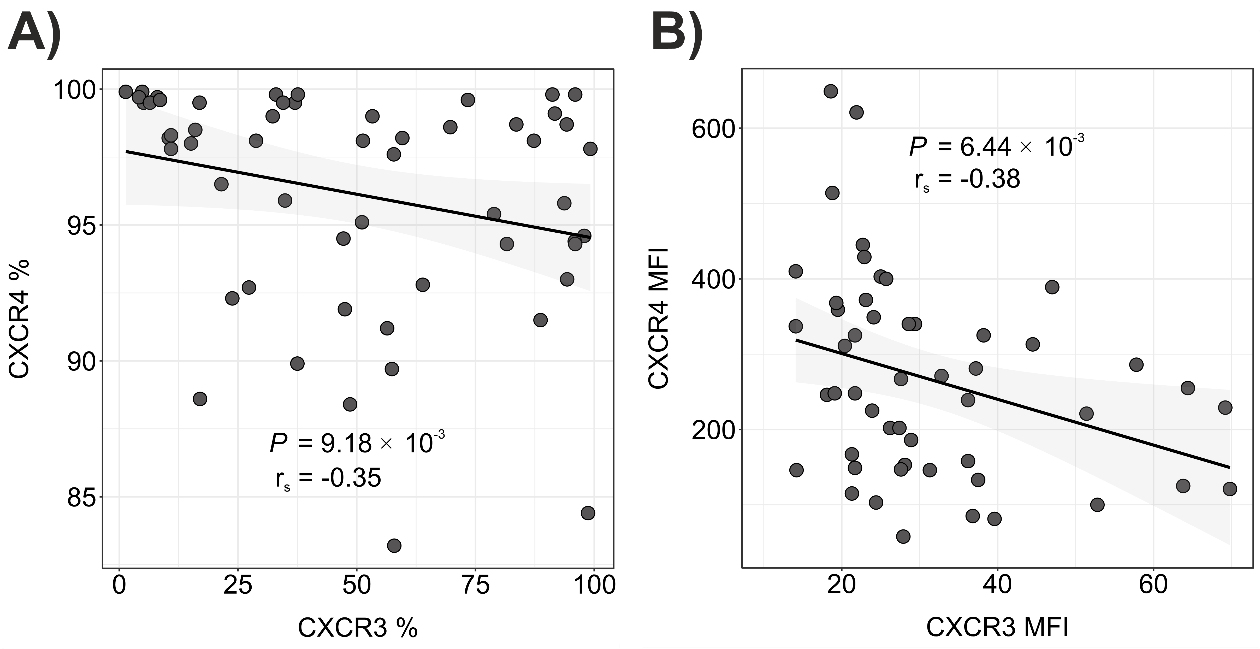
**

**Figure S4. (A)** Patient similarity network based on levels of CXCR3, CXCR4, CRCR5, and CCR7, and **(B)** distribution of chemokine expression in individual clusters. In CD5^low^ subpopulation, four patient clusters were formed (L1-L4), in CD5^high^ five clusters were formed (H1-H5). Individual clusters are coloured; each dot corresponds to one patient; the lines connect the patients with the highest similarity in chemokine profiles. the y-axis in the graphs represents the expression for each chemokine normalized to the maximum value in the data set.

**
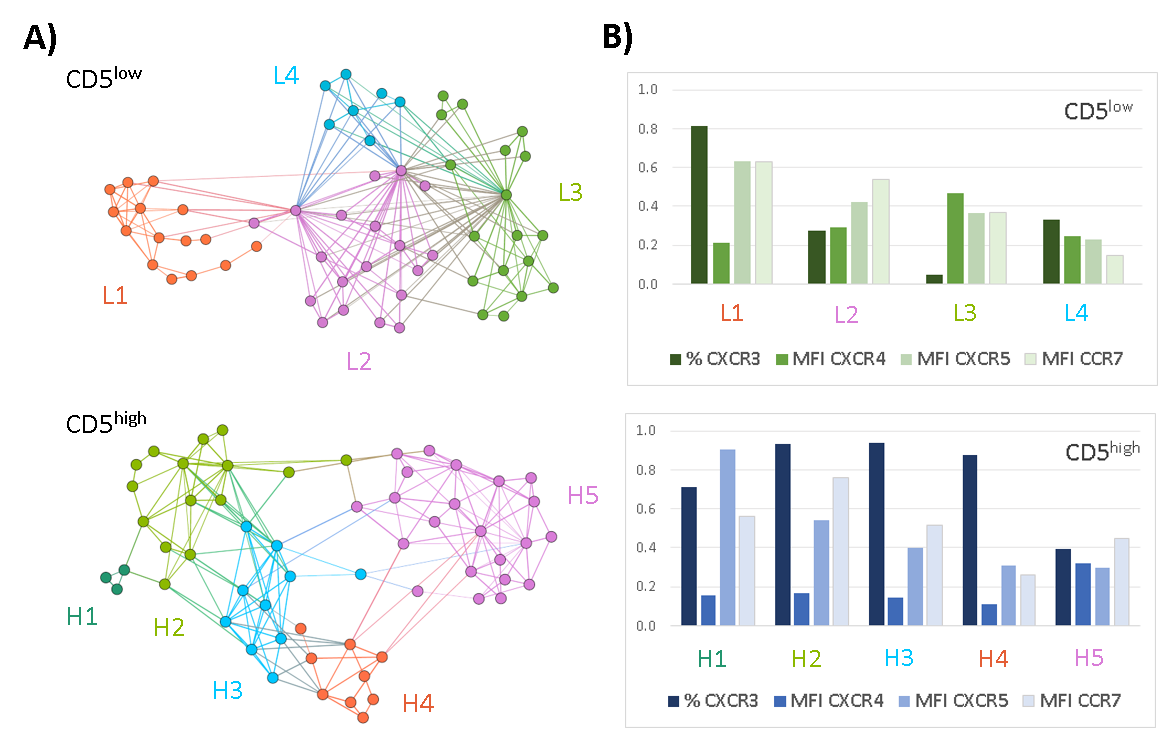
**
